# Endurance training remodels sperm-borne small RNA expression and methylation at neurological gene hotspots

**DOI:** 10.1186/s13148-018-0446-7

**Published:** 2018-01-25

**Authors:** Lars R. Ingerslev, Ida Donkin, Odile Fabre, Soetkin Versteyhe, Mie Mechta, Pattarawan Pattamaprapanont, Brynjulf Mortensen, Nikolaj Thure Krarup, Romain Barrès

**Affiliations:** 1 The Novo Nordisk Foundation Center for Basic Metabolic Research, Faculty of Health and Medical Sciences, University of Copenhagen, 2200 Copenhagen, Denmark; 2 Center for Diabetes Research, Gentofte Hospital, University of Copenhagen, Hellerup, Denmark; 3 University of Copenhagen, Blegdamsvej 3B, 2200 Copenhagen, Denmark

## Abstract

Remodeling of the sperm epigenome by lifestyle factors before conception could account for altered metabolism in the next generation offspring. Here, we hypothesized that endurance training changes the epigenome of human spermatozoa. Using small RNA (sRNA) sequencing and reduced representation bisulfite sequencing (RRBS), we, respectively, investigated sRNA expression and DNA methylation in pure fractions of motile spermatozoa collected from young healthy individuals before, after 6 weeks of endurance training and after 3 months without exercise. Expression of 8 PIWI interacting RNA were changed by exercise training. RRBS analysis revealed 330 differentially methylated regions (DMRs) after training and 303 DMRs after the detraining period, which were, in both conditions, enriched at close vicinity of transcription start sites. Ontology analysis of genes located at proximity of DMRs returned terms related to neurological function at the trained state and, to a much lesser extent, at the detrained state. Our study reveal that short-term endurance training induces marked remodeling of the sperm epigenome, and identify genes related to the development of the central nervous system as potential hot spots for epigenetic variation upon environmental stress.

**Electronic supplementary material:**

The online version of this article (10.1186/s13148-018-0446-7) contains supplementary material, which is available to authorized users.

## Introduction

Gametes contain epigenetic information that plays a fundamental role in embryonic development [1]. Any pre-conceptional disturbance of the gametic epigenome has thus the potential to alter the phenotype of the next generation offspring through so-called epigenetic inheritance. In spermatozoa, not only methylation of DNA and post-translational modifications of histones carry epigenetic signals but also, sperm-borne small RNA (sRNA) can contribute to epigenetic inheritance, as supported by microinjection experiments of spermatozoal RNA into fertilized oocytes [2–4].

Epidemiological evidence indicates that paternal nutrition before conception can alter the phenotype of the following generation [5, 6]. Numerous animal studies have now clearly established that environmental factors can remodel the sperm epigenome at the level of post-translational modifications of histones, DNA methylation, and sRNA [7–14]. Previously, we showed that sRNA expression and DNA methylation are altered in sperm cells of obese men after gastric-bypass-induced weight loss [15]. Whether physical exercise, a potent intervention to treat or prevent obesity and related diseases like obesity and type 2 diabetes (T2D) [16, 17], can concomitantly remodel the sperm sRNA and DNA methylation profile is unknown.

Here, we hypothesized that endurance training changes the epigenetic profile of human spermatozoa. We used an intervention protocol of exercise training and detraining, to dissociate the effect of exercise to that of time, and to analyze the potential long-term memory of endurance training on the sperm epigenome. We show that exercise training specifically remodels the expression of several sRNAs and changes DNA methylation at specific gene hotspots related to brain function and development.

## Results and discussion

Single ejaculates were obtained at three different time points; at baseline (referred to as *Untrained*), 4 days after ending a six-week endurance exercise intervention (referred to as *Trained*) and after 3 months without exercise training (referred to as *Detrained*). Analysis of DNA methylation was performed on the 12 participants while for sRNA, a subset of 6–9 participants was analyzed (see method section for details, see Additional file 1: Figure S1 for an overview). Clinical characteristics of the volunteers at the three time points are presented in Table 1A and B. As expected, aerobic capacity, as measured by VO_2_ peak, was increased from a median value of 44.15/46.2 (respectively for the DNA methylation/sRNA subsets) ml/kg/min at the untrained state to 56.2/53.7 ml/kg/min at the trained state (*p* < 0.001/*p* < 0.005). Three months after the training program, VO_2_ peak decreased to 51.2/50.7 ml/kg/min, and was no longer significantly higher than baseline. We did not observe any inter-individual differences in sperm quality throughout the course of the exercise intervention, nor the detraining period.

**Table 1:** Clinical characteristics of subjects at the *Untrained*, *Trained*, and *Detrained* state

A			
* Cohort used for DNA CpG analyses, n = 12*			
	Untrained	Trained	Detrained
Age – years	22 (18 a 27)	23 (18 a 27)	23 (19 a 28)
Weight - kg	79.0 (63.7 a 105.8)	77.5 (63.7 a 102.2)	79.1 (62.6 a 105.3)
Body mass index - kg/m^2^	22.8 (19.5 a 27.6)	22.9 (19.6 a 26.7)	23.1 (19.2 a 27.4)
Waist – cm	87 (79 a 90)	79* (73 a 87)	80.8^*#*^ (72.6 a 92.6)
Hip – cm	93 (88 a 104)	89* (82 a 99)	90^*#*^ (82 a 101)
Waist/Hip	0.92 (0.88 a 0.99)	0.88 (0.83 a 0.96)	0.91 (0.86 a 0.97)
VO_2_	2780 (3585 a 4469)	4440* (3319 a 5793)	3971 (2763 a 4766)
VO_2_/kg	44.1 (39.7 a 53.9)	56.2* (48.4 a 65.2)	51.2 (38.3 a 55.5)
Glucose (fasting) – mmol/l	4.8 (4.3 a 5.4)	4.8 (4.4 a 5.7)	4.7 (4.3 a 5.3)
Insulin – pmol/l	55 (31 a 106)	51 (25 a 117)	57 (27 a 80)
HOMA-IR	1.85 (1.11 a 4.03)	1.9 (0.9 a 4.9)	1.9 (0.9 a 2.9)
HbA1c - %	32 (26 a 38)	33 (28 a 36)	31^*#*^ (23 a 36)
Plasma cholesterol (total) – mmol/l	4.2 (3.4 a 5.6)	4.0 (3.0 a 4.8)	4.3 (3.4 a 5.4)
Low density lipoprotein – mmol/l	2.3 (1.9 a 3.3)	2.4 (1.8 a 3.0)	2.6 (1.8 a 3.4)
High density lipoprotein – mmol/l	1.4 (1.0 a 1.7)	1.4 (1.1 a 1.6)	1.3 (1.0 a 1.8)
Triglyceride – mmol/l	1.0 (0.6 a 1.5)	0.9 (0.6 a 1.7)	0.9 (0.5 a 1.8)
C-reactive protein – mg/l	1.0 (1.0 a 3.0)	1.0 (1.0 a 1.0)	1.0 (1.0 a 2.5)
Leukocytes – × 10^9^/l	6.0 (3.3 a 7.1)	5.4 (4.3 a 6.7)	5.4 (4.1 a 6.5)
B			
* Cohort used for sRNA analyses, n = 6*			
	Untrained, *n* = 6	Trained, *n* = 6	Detrained, *n* = 6
Age – years	23 (18.1 a 26.9)	23 (18.1 a 26.9)	24 (19 a 27)
Weight - kg	79.8 (64.4 a 88.5)	79.8 (64.3 a 86.2)	81.2 (63.0 a 89.7)
Body mass index - kg/m^2^	22.8 (19.8 a 24.8)	22.7 (19.8 a 24.1)	22.9 (19.4 a 24.0)
Waist – cm	88.5 (78.6 a 95.2)	79.2* (72.4 a 88)	80.5 (72.4 a 93.9)
Hip – cm	92 (88.2 a 99.8)	90.2 (85.4 a 97.2)	91 (83 a 99)
Waist/Hip	0.9 (0.9 a 1)	0.9 (0.8 a 0.9)	0.91 (0.83 a 0.95)
VO_2_	3736 (2766 a 4460)	4221 (3459 a 5167)	3830 (2766 a 4621)
VO_2_/kg	45 (40.1 a 53.8)	52.6 (48.1 a 63.5)	50.7 (37.4 a 55.5)
Glucose (fasting) – mmol/l	4.8 (4.3 a 5.4)	4.8 (4.6 a 5.8)	4.7 (4.4 a 5.3)
Insulin – pmol/l	80 (54.4 a 107.5)	56 (41.4 a 121.4)	58 (45 a 81)
HOMA-IR	2.9 (1.8 a 4.1)	2 (1.5 a 5.2)	2.0 (1.7 a 2.9)
HbA1c - %	33.5 (29.2 a 38.8)	33.5 (28.1 a 36)	30^**#**^ (26 a 37)
Plasma cholesterol (total) – mmol/l	4.6 (3.9 a 5.8)	4.1 (3.6 a 4.8)	5.1 (4.3 a 5.8)
Low density lipoprotein – mmol/l	2.6 (2.1 a 3.6)	2.5 (1.9 a 3.2)	1.4 (1.0 a 1.9)
High density lipoprotein – mmol/l	1.3 (1 a 1.5)	1.4 (1 a 1.5)	2.9 (2.4 a 4.1)
Triglyceride – mmol/l	1.2 (0.9 a 1.6)	1.1 (0.7 a 1.7)	1.1 (0.8 a 1.9)
C-reactive protein – mg/l	1 (1 a 2.8)	1 (1 a 1)	1.0 (1.0 a 2.9)
Leukocytes – × 10^9^/l	6.1 (3.4 a 8)	6.1 (4.2 a 7.7)	5.4 (4.4 a 11.2)

A Subjects studied for DNA methylation. B Subjects studied for sRNA expression. HOMA-IR: Homeostatic Model Assessment of Insulin Resistance. Results are medians with 2.5 and 97.5 percentiles. Differences between time points were determined using a paired *t* test or a Wilcoxon signed rank (Insulin and HOMA-IR in both tables and Triglyceride in Table 1a) and are Holm-Bonferroni corrected. Age and C-reactive protein were not tested. *P < 0.05 *Untrained* vs. *Trained*, ^**#**^*P* < 0.05 *Untrained* vs. *Detrained*

### Exercise training modulates sperm sRNA expression in a reversible fashion

**Fig. 1:**
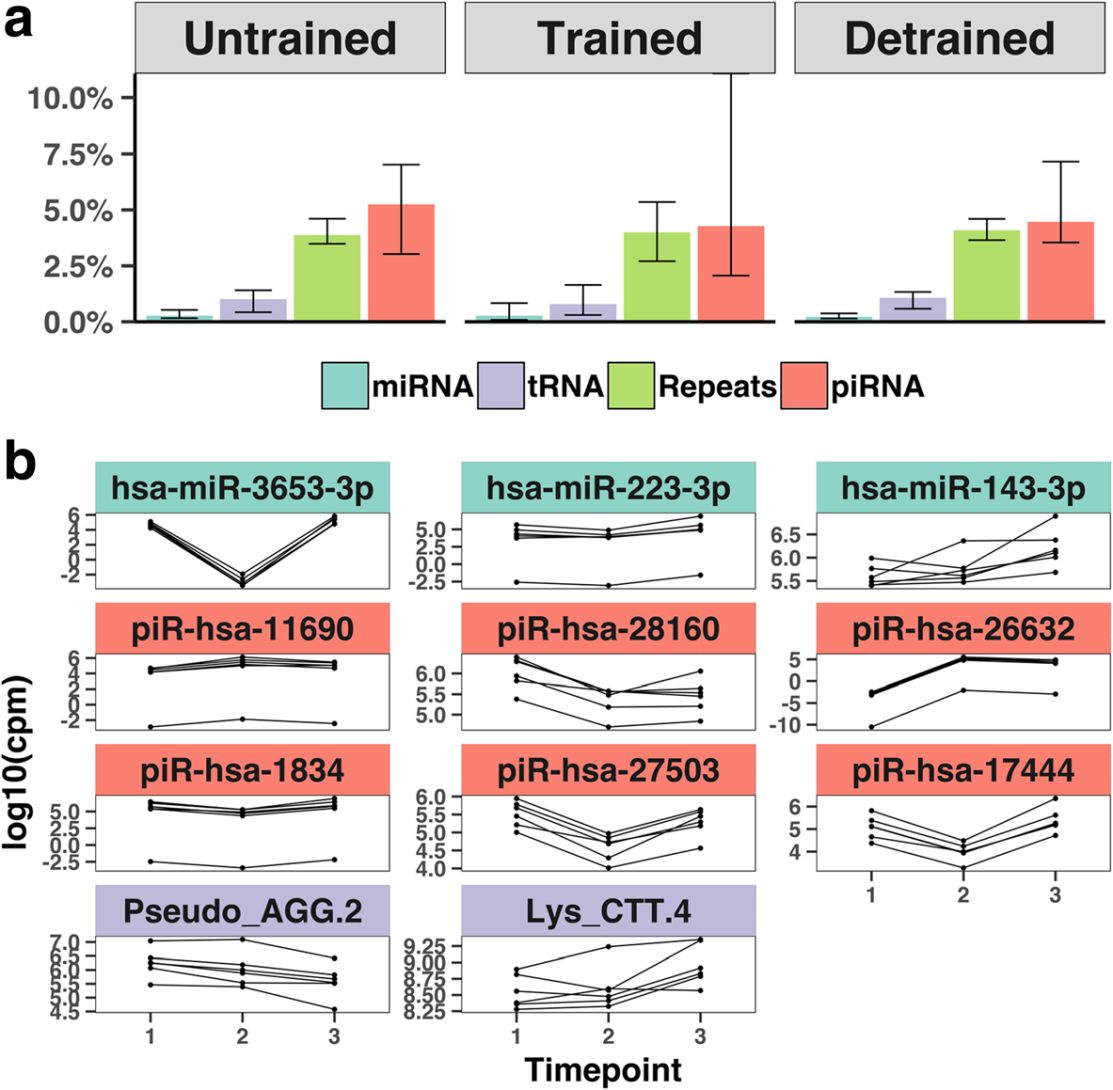
Effect of endurance training and detraining on sperm-borne small non-coding RNA and subsets of repetitive elements. **a** Median abundance of selected sRNA subtypes at the three different time points. Median error bars are from lowest to highest observation from sRNA-seq data. **b** The expression levels of selected subsets of sRNA (miRNA, green; tRNA, red; piRNA, purple) are presented at the three different time points for each individual. Data are presented as log-transformed sequence reads per million (1 = Untrained, 2 = Trained, 3 = Detrained)

We first investigated small RNA (sRNA) expression in purified sperm from the same subjects at the *Untrained*, *Trained* and *Detrained* state by sRNA sequencing. Consistent with previous reports, PIWI-interacting RNAs (piRNA) are expressed at higher levels than tRNA fragments (tRF) and microRNAs in human sperm [15, 18] (Fig. 1a, Additional file 2: Figure S3, Additional file 3: Figure S4, Additional file 4: Figure S5). To analyze the effect of training on sRNA expression, we identified 5 piRNAs and 27 fragments of repetitive elements that were differentially expressed between the *Trained* and the *Untrained* state. Between any two time-points, a total of 3 miRNAs, 2 tRNAs, 6 piRNAs, and 38 repetitive elements were differentially expressed (Fig. 1b, Additional file 5: Figure S6), false discovery rate [FDR] < 0.1; Additional file 6: Tables S1–S4, Additional file 7: Table S5). To identify reversible sRNA expression changes, we compared the changes observed between *Untrained*/*Trained* with the changes at the *Trained/Detrained* and *Untrained/Detrained*. We found several piRNAs underwent a transient change in expression (Fig 1b). The expression of five of the six piRNA was changed between the *Untrained* and *Trained* state, while no piRNA was differentially expressed when comparing the *Untrained* and *Detrained* state, indicating a specific response to exercise training. On the other hand, no miRNAs were differentially expressed between the *Untrained* and *Trained* state, but one out of three were found between *Untrained* and *Detrained* and two of three were found between *Trained* and *Detrained*, suggesting changes in miRNA expression are not primarily triggered by exercise training. Expression of tRNAs followed a similar pattern where all changes were exclusively detected when comparing the *Untrained* to the *Detrained* state. Lastly, repetitive elements did not follow a specific pattern (Additional file 8: Figure S2). Taken together, our results suggest exercise induces acute response in piRNA expression, which is reverted after cessation of training. Expression of miRNA and tRNA, however, seems to be more stable with time.

In silico target prediction for piR-hsa-28,160 returned multiple copies of the ILF3/NF90-interacting RNA *Small ILF3/NF90-associated RNA (SNAR),* a regulator of the let7 family member let7a, a miRNA family well documented in inflammation, glucose metabolism [19–23] and, more recently, epigenetic inheritance [11]. Of the remaining piRNAs, one has no predicted target, one targets FAM225A and B, two ncRNAs with no known function which are highly expressed in testis and one targets NSD1, a transcription factor that has been associated with Sotos syndrome [24], the symptoms of which include mild intellectual impairment and co-occur with autism [25]. The remaining two piRNAs target small nucleolar RNAs (snoRNAs). Both of those snoRNAs are predicted to regulate ribosomal RNA (rRNA) maturation. snoRNAs have been implicated in both cancer and lipotoxicity, and thought to exert miRNA-like function, notably for the regulation of alternative splicing [26, 27]. It was previously shown that a loss of snoRNA leads to a variety of diseases, such as the Prader–Willi syndrome, which is characterized by morbid obesity and intellectual impairment [28]. Thus, it is possible that changes in the expression of sperm-borne snoRNAs after endurance training influences the developmental programming of the embryo and predispose/protect from disease. Altogether, our data demonstrate that sperm-borne sRNA content can be dynamically affected by a 6-week endurance training intervention. The functional relevance of the exercise-induced sRNA differential expression in human sperm on the developmental programming of the embryo remains to be investigated.

### Exercise training remodels methylation of brain genes

**Fig. 2:**
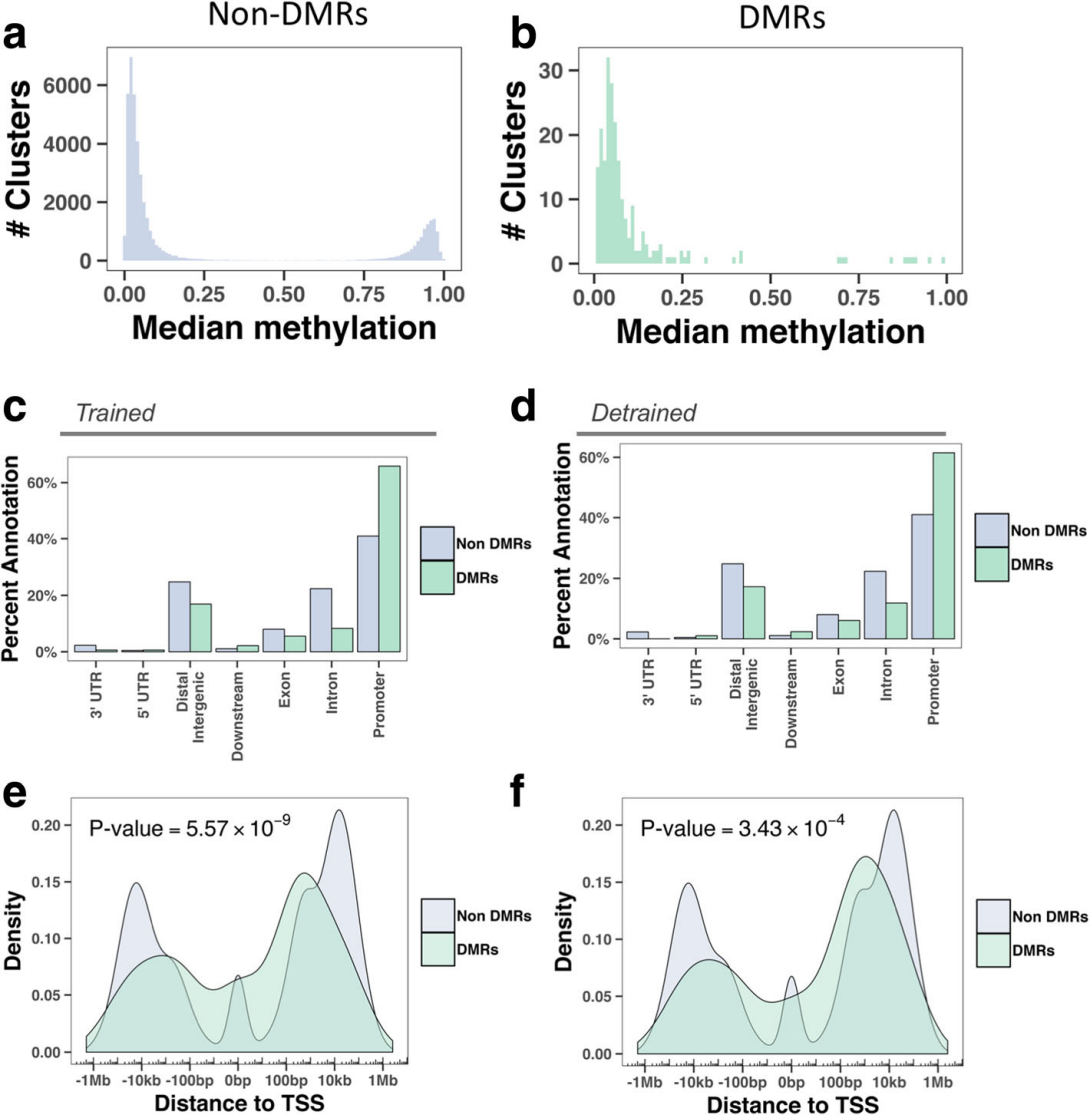
Differential methylation after training is enriched at transcription start sites. **a**, **b** Median methylation levels for each cluster at non-DMR regions (**a**) compared to DMR regions (**b**). **c**, **d** Distribution of annotations comparing non-DMRs and DMRs on a percent scale. Visualized are both the Untrained/Trained (Trained, c) and Untrained/Detrained (Detrained, d) comparisons. Relative enrichment is visualized by the difference in bar height between DMRs and non-DMRs. **e**, **f** Density of transcription start sites (TSS) distances for DMRs and non-DMRs for the Untrained/Trained (Trained, e) and Untrained/Detrained (Detrained, f) comparisons. The DMRs come from a different distribution than the non-DMRs, as shown by a Kolmogorov–Smirnov test (*P* value on the plots)

To investigate the effect of exercise training on DNA methylation, we performed Reduced Representation Bisulfite Sequencing (RRBS) on the pure sperm fractions collected at each time point. In total, 119,624 CpG clusters covering more than 1.4 million individual CpGs were interrogated by the RRBS protocol. Results were analyzed using a FDR 5 or 10% cut-off (Additional file 7: Table S6). With a FDR 10% cut-off, compared to the *Untrained* state, the *Trained* state returned 330 differentially methylated regions (DMRs), while 303 DMRs were detected 3 months after the last training session of the training program (at the *Detrained* state). With a 5% FDR cut-off, we found 177 DMRs at the Trained and 190 DMRs at the *Detrained*. Analysis of median methylation showed that, while the clusters investigated followed a bi-modal distribution of low or high methylation, the DMRs were almost entirely located in a low methylated context (Fig. 2a). In both the *Trained* and *Detrained* state, DMRs were enriched at promoter regions over exon, intron and distal intergenic regions (Fig. 2b, c). Closer analysis of promoter regions revealed that DMRs were most preferably located in a 10 kb region centered on the transcription start site (TSS) (Fig. 2d, e). Collectively, these data show that DNA methylation changes in response to exercise occur at specific genomic elements, and strongly suggests a role in the control of transcription initiation.

**Fig. 3:**
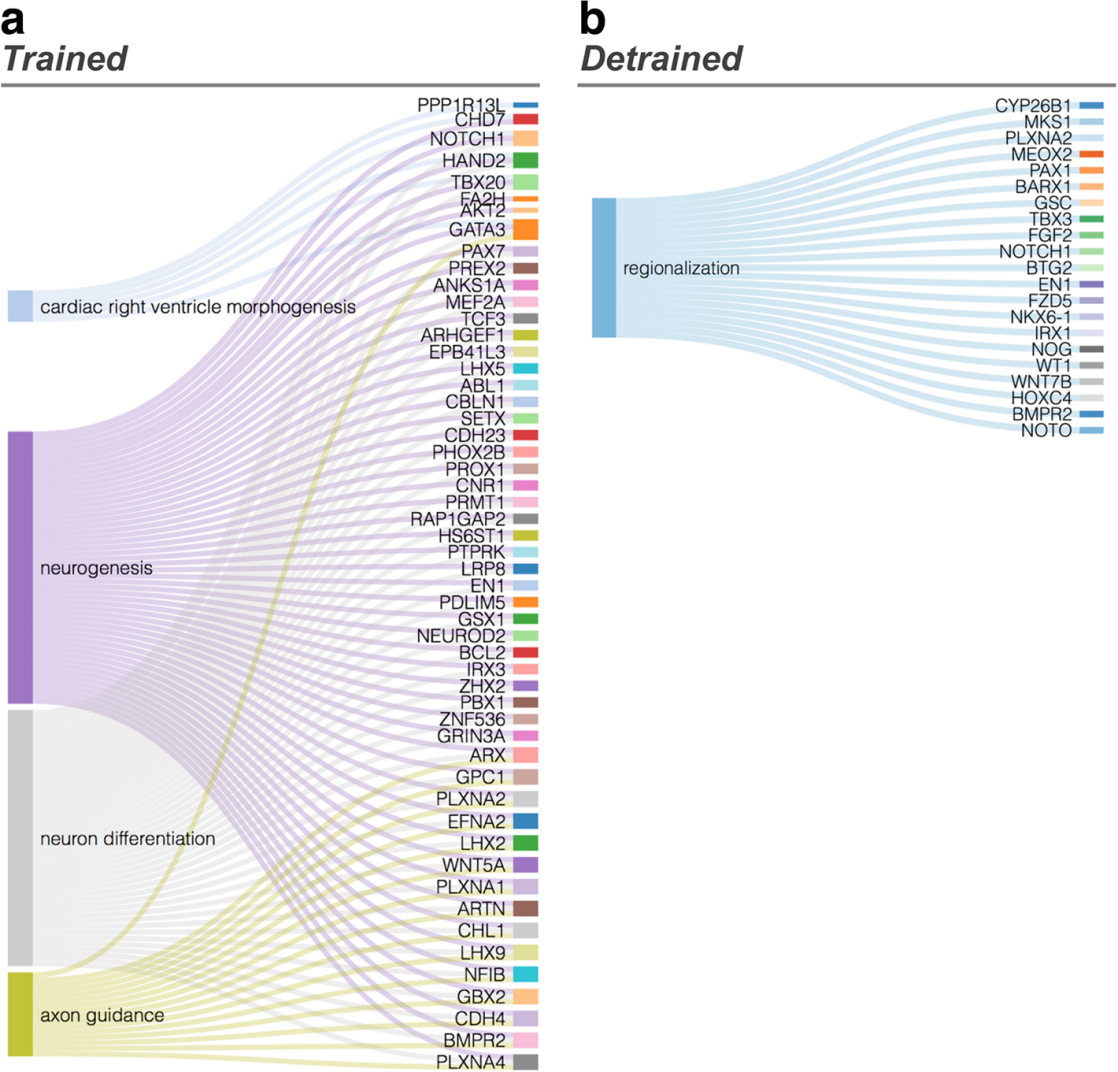
Higher enrichment for terms related to neurological development and function after training compared to 3 months after training. Sankey diagram showing genes at proximity of the Trained state DMRs (**a**) or Detrained state DMRs (**b**) and related to development. Gene symbols are shown

To gain insight into the functional relevance of differential methylation after exercise, we performed a gene ontology analysis for the genes proximal to exercise DMRs at 5 or 10% FDR using g:Profiler [29]. Regardless of the FDR cut-off, at the *Trained* state, significant enrichment was found for the ontology terms related to the development of the central nervous system such as “neurogenesis”, “neuron differentiation”, and “axon guidance” (Additional file 9: Tables S7 and S8 and Fig. 3a). It is noteworthy that the gene ontology term “neurogenesis” contains all genes of the aforementioned terms, except the gene *PPP1R13L*. While some DMRs survived 3 months after training, gene ontology analysis of the *Detrained* state only returned the more generic term “regionalization” (Fig. 3a). Study design of published intervention studies (including from our group) did not establish if epigenetic variation in sperm is simply time-related or specifically triggered by the intervention itself [15, 30]. Here, the loss of both ontology term specificity and number at the *Detrained* state infers that exercise triggers specific DNA methylation changes and that these changes are not caused by simple time-related effect.

Genes related to the development of the central nervous system were previously identified as genes escaping epigenetic reprogramming in human primordial germ cells and early embryogenesis [31]. While we did not find, using a hyper-geometric test, a statistically significant overlap between our exercise-responsive DMRs and regions escaping epigenetic reprogramming during human gametogenesis [31], we found seven genes proximal to our DMRs in common; *SMCO1*, *PCDH10*, *FAM160A1*, *TRIML1*, *ABL1*, *SETX*, and *TSPY3* (see Table 2). Testing for overlap between our gene list and past reports investigating sperm DNA methylation changes in health and disease, we also did not detect specific enrichment between genes at proximity of our exercise DMRs and genes reported in sperm from obese [15], from an autism cohort [32] or after 3 months of endurance training [30]. These results may imply that while genes related to the development of the central nervous system are epigenetically susceptible to environmental influences in male gametes, each environmental insult triggers changes on a specific subset of genes. Alternatively, the difference in regions covered by each of these studies could explain the lack of overlap across cohorts. Nevertheless, our results strengthen that a subset of genes involved in the development of the central nervous system represents a genomic hotspot for epigenetic variation under environmental influences that has potential to convey reprogramming signals to the embryo.

**Table 2:** Selected DMRs at the trained state. DMRs which are found to be responsive to exercise and whose nearest gene has been found to escape epigenetic reprogramming, Chr is chromosome, Difference is the median of methylation differences observed within the cluster, upon exercise training

Chr	Start	End	Difference	Distance to TSS	Gene Symbol	Description
chr3	196,260,561	196,260,561	− 5.3%	− 18,324	SMCO1	Single-pass membrane protein with coiled-coil domains 1
chr4	132,671,876	132,671,876	2.8%	− 1,398,594	PCDH10	Protocadherin 10
chr4	152,283,426	152,283,475	− 15.8%	− 46,923	FAM160A1	Family with sequence similarity 160 member A1
chr4	189,100,182	189,100,182	− 30.9%	35,385	TRIML1	Tripartite motif family like 1
chr9	133,616,767	133,616,767	− 13.0%	27,499	ABL1	ABL proto-oncogene 1, non-receptor tyrosine kinase
chr9	135,127,959	135,128,001	− 47.2%	77,885	SETX	Senataxin
chrY	9,384,788	9,384,795	12.8%	19,299	TSPY3	Testis specific protein, Y-linked 3

**Fig. 4:**
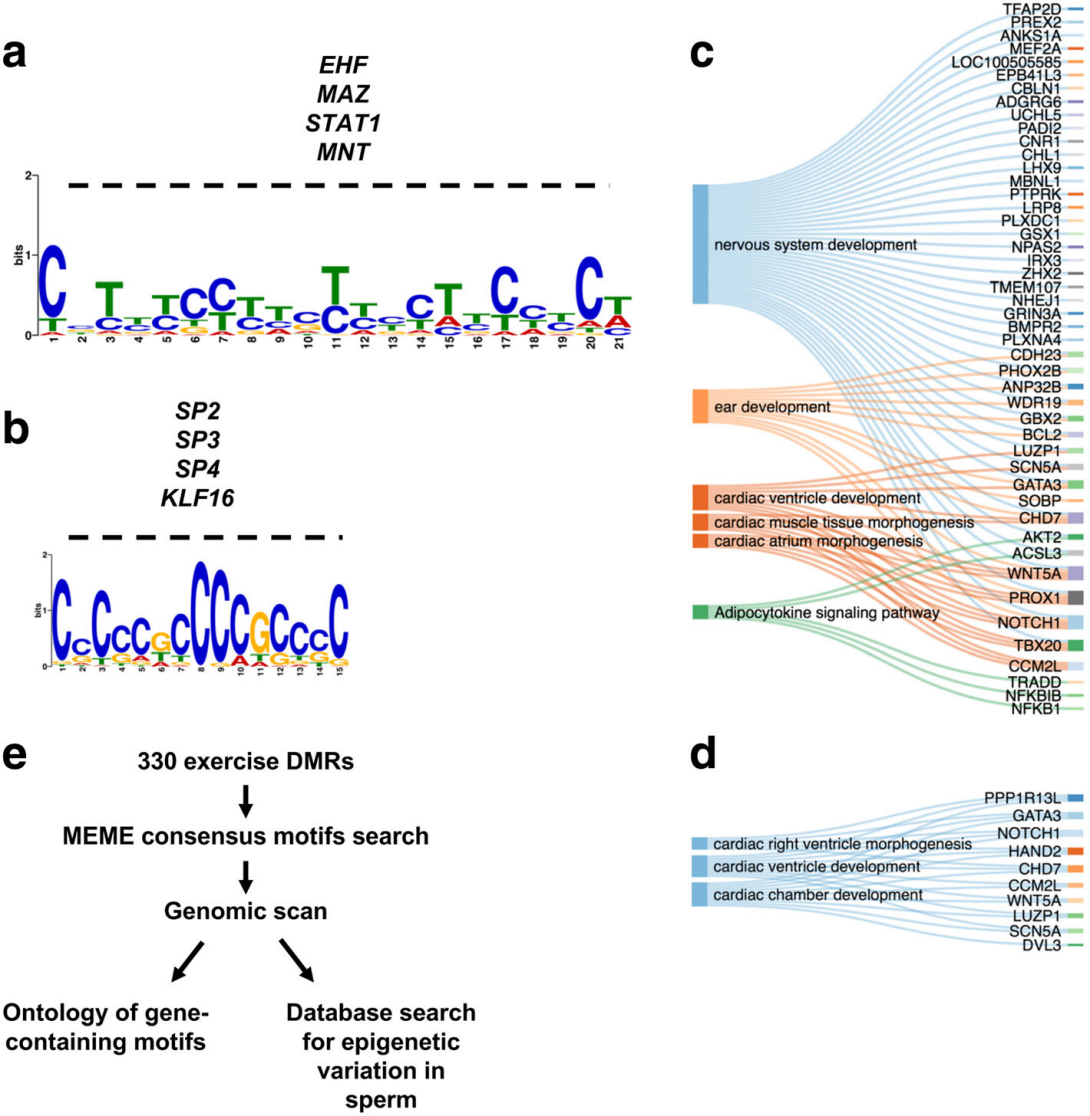
Consensus sequence analysis of the DMR reveals transcription factor binding sites at proximity of genes related to neurological development and function. **a**, **b** Consensus sequences found in the 330 exercise DMRs from both *Trained* and *Detrained* states, as predicted using the MEME tool. **c**, **d** Sankey diagrams representing the gene ontology analysis for genes containing the consensus sequence respectively identified in **a** and **b**. **e** Short description of computational analysis pipeline

To identify if epigenetically variable genes in sperm carry common sequence features, we searched for motifs surrounding the exercise DMRs found in a low methylation context using the MEME suite [33]. We discovered that two motifs cover the majority of DMRs (96% of DMRs contained at least one motif, and 64% contain both). Prediction of transcription factor binding returned putative binding site for the transcription factors *EHF*, *MAZ*, *STAT1*, and *MNT* (Fig. 4a), and SP2, SP3, SP4, and KLF16 for the respective motifs. Most importantly, a genomic scan for genes containing each respective motif returned that genes containing the motif of Fig. 4a were enriched for the term “nervous system development” (Fig. 4c) while motif of Fig. 4b did not (Fig. 4d). This observation indicates that we identified motifs located at proximity of epigenetically-variable genes and reinforces our finding that epigenetically variable genes in human sperm relate to the development of the central nervous system.

This study contains a few limitations worth noting. The sample size is relatively small (*n* = 6 for sRNA and *n* = 12 for RRBS), due to a technical limitation when extracting DNA and RNA from the on single ejaculate, and in all three time-points (Untrained, Trained, and Detrained). However, the fact that each participant were assessed at all of the three time points considerably increases statistical power [34]. The lack of a control group accounting for time-related epigenetic alterations during the 6 week of exercise intervention can be seen as a limit to this study; however, others have reported no significant alterations in total methylation levels in spermatozoa of a control, non-exercising group, in a 3-month sampling interval [30].

In conclusion, our data provide evidence that endurance training remodels sRNA expression and the DNA methylation profile at close proximity of transcription start sites, specifically, at genes related to neurological development and function. These findings highlight the dynamic nature of the spermatozoal epigenome in response to environmental or lifestyle factors in humans. Future studies will determine the role of environmentally induced epigenetic changes in sperm on the development of the embryo and phenotype of the offspring.

## Material and methods

### Subjects and sample collection

The study was approved by the Ethics Committee from the Capital Region of Denmark (reference H-1-2013-064) and informed consent was obtained from all participants. A portion of this cohort has previously been described [35]. Clinical characteristics of participants at all three time-points are presented in Table 1A and B. For the analysis of sRNA nine participants were analyzed at the untrained and trained, six participants were analyzed at the detrained state. Analysis of DNA methylation was performed on 12 participants at all three time points. All recruited participants were young, healthy, sedentary Caucasian males in their reproductive age (18–35 years). Exclusion criteria were regular smoking, alcohol consumption of > 14 units per week, presence of chronic or acute disease as well as daily intake of medicine. Men exercising more than twice per week, or who within the last 2 years had performed exercise on competitive levels, were excluded. VO_2max_ tests were performed by incremental exercise to volitional fatigue on an electromagnetically braked cycle ergometer (Monark Ergomedic 839E, Sweden) under fasting conditions. Pulmonary gas exchange was measured during the test breath-by-breath with a gas analyzing system (Oxycon Pro, Jaeger, Germany). All participants were fecund and cleared for testicular and andrological abnormalities by inspection, anamnesis, and palpation. Microscopy was used to rule out spermatozoal morphological abnormalities and to count sperm concentration. Semen samples were delivered by masturbation after an overnight fast and a period of minimum 3 and maximum 7 days of ejaculative abstinence. Ejaculates were immediately stored at 37 °C. Venous blood was drawn at fasting conditions.

### Exercise intervention

Before the exercise intervention, all participants delivered a semen sample, blood sample and performed a VO_2max_ test (*Untrained*). The 6-week exercise program was performed by five weekly 1-h sessions for 6 weeks with supervised spinning classes by a certified instructor. The spinning classes were kept at an intensity of 70% of the participants’ individual reserve capacity of their max pulse, determined by a max test performed before the exercise intervention. All participants participated in all sessions within the 6 weeks, and performance at each session was in accordance with the required intensity, as monitored individually by personal pulse monitors. After the 6-week exercise intervention, participants rested for 4 days before delivering the *Trained* ejaculate and performing VO_2_max test. Participants returned to their habitual *Untrained* exercise level for the following 3 months until their last session of semen sample delivery, blood sampling and VO_2_max were performed (*Detrained*). Compliance with the detraining program was verified by self-reporting at regular check-ups by investigator.

Changes in clinical parameters were only analyzed on respective subset of participants included for DNA methylation or sRNA expression. Thus, sRNA was calculated based on data from six participants. Tests for the RRBS cohort was based on 12 participants. Clinical parameters were tested for normality using a Shapiro-Wilk test, *p* values for normally distributed parameters were calculated with a paired t-test, while non-normal variables were tested with a Wilcoxon signed rank test. All *p* values were corrected for family-wise error rate by the Holm–Bonferroni method.

### Isolation of motile spermatozoa

A “swim-up” procedure was performed to exclude somatic cells and to isolate motile spermatozoa, which resulted in the isolation of the spermatozoa with the highest fertilization potential: 0.5 ml of semen was overlaid with 2 ml of medium (Earle’s Balanced Salt Solution (Sigma) with 3.2 mg/ml Human Serum Albumin (Sigma) and 25 mM Hepes) in round-bottom tubes and incubated at 37 °C at a 45° angle for 2 h. The upper fractions were pooled per ejaculate, and the spermatozoa counted by microscopy. The potential presence of somatic cells was inspected under a photon microscope.

### sRNA-Seq and RRBS

Total RNA was isolated by the TRIzol® method (Life Technologies) from the sperm cells of six men before and after the 6-week exercise intervention, and after 3 months of detraining. The sRNA libraries were prepared using the NEBNext® Multiplex Small RNA Library Prep Set for Illumina (New England Biolabs), according to the manufacturer’s instructions. Molecules of 20–50 nucleotides were separated by acrylamide gel electrophoresis, extracted, and sequenced on a HiSeq2500 Illumina instrument as 50 bp single-end reads, and processed by CASAVA 1.8.2.

The filter for including a feature in the sRNA analysis was dependent on the type of feature being analyzed. Due to the vast differences in sequencing depth of the different sRNA types, a single count per million (CPM) cutoff would have either included features with almost no reads or excluded features with many reads. Instead, a dynamic cutoff was used that depended on the total number of reads for that feature. The formula used to calculate the cutoff was:

$$ cutOff=5\times median\left(\frac{n_{reads}}{10^6}\right) $$

Where *n*_*reads*_ is a vector containing the total number of reads assigned to this type of sRNA for each sample. This translates to a cut-off of 0.2 for miRNA, 0.5 for tRNA, 2.7 for piRNA and 2.9 for Repetitive Elements (Additional file 6: Tables S2–S4, Additional file 7: Table S5). Features with more than the cut-off in 1/3 or more of the samples (eight or more samples) was included in the test for differential expression.

For DNA methylation analysis, genomic DNA was extracted from the sperm cells of 12 men before and after the exercise intervention, as well as after the detraining period, with the Nucleon™ BACC Genomic DNA Extraction Kit (GE Healthcare, Life Sciences). The protocol was modified for processing of sperm, according to the manufacturer’s recommendations. Reduced Representation Bisulfite Sequencing libraries were constructed as previously described [15]. Briefly, 200 ng of genomic DNA was digested with 40 U of *MspI* enzymes (New England Biolabs) and ligated to TruSeq (Illumina) sequencing adaptors. Bisulfite conversion was conducted once with the EpiTect bisulfite kit (Qiagen) in accordance with the manufacturer’s instructions, and the converted DNA was amplified by PCR and sequenced on a HiSeq2500 Illumina instrument as 50 bp single end reads, and processed by CASAVA 1.8.2.

### Analysis of sequencing data

sRNA reads were aligned to hg19 using the subread aligner using the recommended settings for miRNA mapping with the exception that only uniquely mapping reads were kept [35]. Unmapped reads were aligned first ribosomal sequences allowing for up to 10 mappings per read, reads that were still unmapped were aligned to miRNA-, piRNA-, tRNA- and repeatmasker-sequences in that order, keeping only reads that could be uniquely mapped. Reads that mapped to the genome in the first step were counted using Feature Counts [36], assigning reads to the feature they overlapped the most, and added to the counts generated in the subsequent mapping steps. Features were filtered for low expression using a dynamic filter, see above, prior to testing for differential expression. Detection of differentially expressed sRNAs was calculated by edgeR and included both participant and training effects. Features with a false discovery rate less than 0.1 were deemed significant. Human mature miRNAs and their precursor sequences were obtained from miRBase [37] version 20. piRNA sequences were downloaded from piRBase version 1.0 [38]. Human tRNAs were retrieved from UCSC genome browser [39]. piRNA targets were predicted using piRNAQuest [40] which uses sequence similarity to predict piRNA targets. Biotype composition of sRNA is visualized as barplots with a height equal to the median and error bars extending from the minimum to maximum observation. A plot of a PCA analysis based on the sRNA composition is available in Additional file 10: Figure S7.

Preprocessing of RRBS reads were done with Trim Galore v0.4.0 & Cutadapt v1.8.3 using the --rrbs flag. Bismark v0.14.4 [41] was used for aligning the reads to the hg19 genome and for computing the CpG coverage. The BiSeq package v1.10 [42] calculated methylation levels and found DMRs. Standard settings were used, except for the function: “clusterSites”, where the settings perc.samples = 0.5 and min.sites = 5 were used. The model formula used was ~ Condition + Patient | Condition. A FDR cutoff of 0.1 was used for selecting the final DMRs, additionally a more stringent cut-off of 5% FDR coupled with a minimum methylation change of 5% and 10% were tested. Gene ontology analysis of nearby genes revealed that similar types of genes were discovered under all levels of stringency. Motif discovery was done using MEME-ChIP [43] on DMRs with a median methylation of less than 20% as the foreground and clusters with the same methylation levels that were not differentially methylated as background. Prior to motif discovery all regions were widened to 500 bp centered on the DMR/cluster. Hierarchical clustering of samples based on estimated methylation is available in Additional file 10: Figure S7. Summary statistics of both sequencing experiments are available in Additional file 11: Table S9. Individual methylation results at each CpG site within DMRs are provided in Additional file 12: Table S10.

Differences between the distribution of distances to a TSS for DMRs and non DMRs were tested using a Kolmogorov–Smirnov test (K-S test). Overlap between the DMRs discovered in this paper and previously reported DMRs were tested with a hypergeometric test (Additional file 13: Figure S8).

## Additional files

Additional file 1: Figure S1. Overview of the experimental setup. (TIFF 1386 kb)

Additional file 2: Figure S3. Median abundance of all sRNA subtypes at the three different time points, height is median error bars are from lowest to highest observation. (TIFF 285 kb)

Additional file 3: Figure S4. Observed abundance of selected sRNA subtypes at the three different time points, columns represent different participants. (TIFF 189 kb)

Additional file 4: Figure S5. Observed abundance of all sRNA subtypes at the three different time points, columns represent different participants. (TIFF 218 kb)

Additional file 5: Figure S6. Boxplot of the expression levels of selected subsets of sRNA (miRNA, green; tRNA, red; piRNA, purple) are presented at the three different time points for each individual. Data are presented as log-transformed sequence reads per million (1 = Untrained, 2 = Trained, 3 = Detrained). (TIFF 973 kb)

Additional file 6: Tables S1 – S4. Differences in sRNA expression profiles in the spermatozoa between the *Untrained*, *Trained* and *Detrained* state. miRNAs, piRNAs, Repetitive Elements, tRNAs and mRNA fragments differentially expressed between *Untrained* and *Trained*, *Trained* and *Detrained*, *Untrained* and *Detrained.* logFC: Log2 Fold Change; logCPM: Log2 counts per million; LR: Likelihood ratio; feature: sRNA name; FDR: False Discovery Rate. (ZIP 1010 kb)

Additional file 7: Tables S5 and S6. Differences in methylation in the spermatozoa between the *Untrained, Trained and Detrained* state. S5 Table shows results for the FDR 10% cut-off and S6 Table shows results for the FDR 5% cut-off. Regions differentially methylated between, *Untrained* and *Trained* as well as *Untrained* and *Detrained.* median.p: median *P*-value in DMR; median.meth.untrained/trained/detrained: median methylation of CpGs in DMR; median.meth.diff: median methylation difference between conditions; annotation: location relative to nearest gene. (ZIP 198 kb)

Additional file 8: Figure S2. The expression levels of selected subsets of Repetitive Elements are presented at the three different time points for each individual. Data are presented as log-transformed sequence reads per million (1 = Untrained, 2 = Trained, 3 = Detrained). (TIFF 1371 kb)

Additional file 9: Tables S7 and S8. Gene ontology analysis of gene located at proximity of the Trained (S6) or the Detrained (S7) DMRs. GO: Gene ontology term. FDR: False Discovery Rate. (ZIP 61 kb)

Additional file 10: Figure S7. PCA plot of the samples based on sncRNA distribution. The three samples 25_2_2, 7_1_2 and 15_1_2 were investigated as possible outliers, but no reason to exclude them could be found. (TIFF 220 kb)

Additional file 11: Table S9. Overview of sequencing results. Number of reads sequenced, number of reads aligned, mapping rate and number of unique aligned reads for the sRNA and RRBS experiment. (XLSX 52 kb)

Additional file 12: Table S10. Detailled DNA methylation results at Differentially Methylated Regions. Sequencing counts are indicated at each CpG for every participants. (XLSX 149 kb)

Additional file 13: Figure S8. Hierarchical clustering of samples based on estimated methylation across all covered CpGs. Sample 17_1 was sequenced less deeply and did not appear to be an outlier. (TIFF 1655 kb)

## Abbreviations

Chr: Chromosome
CPM: Count per million
DMRs: Differentially methylated regions
HOMA-IR: Homeostatic Model Assessment of Insulin Resistance
K-S test: Kolmogorov-Smirnov test
miRNA: Micro-RNA
piRNA: PIWI-interacting RNAs
RRBS: Reduced representation bisulfite sequencing
rRNA: Ribosomal RNA
snoRNAs: Small nucleolar RNAs
sRNA: Small RNA
T2D: Type 2 diabetes
tRF: tRNA fragments

## References

[1] Reik W (2007). Stability and flexibility of epigenetic gene regulation in mammalian development. Nature.

[2] Dias BG, Ressler KJ (2014). Parental olfactory experience influences behavior and neural structure in subsequent generations. Nat Neurosci.

[3] Gapp K, Jawaid A, Sarkies P, Bohacek J, Pelczar P, Prados J (2014). Implication of sperm RNAs in transgenerational inheritance of the effects of early trauma in mice. Nat Neurosci.

[4] Grandjean V, Fourre S, De Abreu DA, Derieppe MA, Remy JJ, Rassoulzadegan M (2015). RNA-mediated paternal heredity of diet-induced obesity and metabolic disorders. Sci Rep.

[5] Kaati G, Bygren LO, Edvinsson S (2002). Cardiovascular and diabetes mortality determined by nutrition during parents' and grandparents' slow growth period. Eur J Hum Genet.

[6] Pembrey ME, Bygren LO, Kaati G, Edvinsson S, Northstone K, Sjostrom M (2006). Sex-specific, male-line transgenerational responses in humans. Eur J Hum Genet.

[7] Carone BR, Fauquier L, Habib N, Shea JM, Hart CE, Li R (2010). Paternally induced transgenerational environmental reprogramming of metabolic gene expression in mammals. Cell.

[8] Barres R, Kirchner H, Rasmussen M, Yan J, Kantor FR, Krook A (2013). Weight loss after gastric bypass surgery in human obesity remodels promoter methylation. Cell Rep.

[9] Chen Q, Yan M, Cao Z, Li X, Zhang Y, Shi J (2016). Sperm tsRNAs contribute to intergenerational inheritance of an acquired metabolic disorder. Science.

[10] Cropley JE, Eaton SA, Aiken A, Young PE, Giannoulatou E, Ho JW (2016). Male-lineage transmission of an acquired metabolic phenotype induced by grand-paternal obesity. Mol Metab.

[11] de Castro Barbosa T, Ingerslev LR, Alm PS, Versteyhe S, Julie Massart J, Rasmussen R, et al. High-fat diet reprograms the epigenome of rat spermatozoa and transgenerationally affects metabolism of the offspring. Molecular Metab. 2015;5(3):184-97. 10.1016/j.molmet.2015.12.002 PMC4770269 26977389

[12] Radford EJ, Ito M, Shi H, Corish JA, Yamazawa K, Isganaitis E (2014). In utero effects. In utero undernourishment perturbs the adult sperm methylome and intergenerational metabolism. Science.

[13] Sharma U, Conine CC, Shea JM, Boskovic A, Derr AG, Bing XY (2016). Biogenesis and function of tRNA fragments during sperm maturation and fertilization in mammals. Science.

[14] Wei Y, Yang CR, Wei YP, Zhao ZA, Hou Y, Schatten H (2014). Paternally induced transgenerational inheritance of susceptibility to diabetes in mammals. Proc Natl Acad Sci U S A.

[15] Donkin I, Versteyhe S, Ingerslev LR, Qian K, Mechta M, Nordkap L, et al. Obesity and Bariatric Surgery Drive Epigenetic Variation of Spermatozoa in Humans. Cell Metab. 2015;23(2):369-78. 10.1016/j.cmet.2015.11.004 26669700

[16] Coffey VG, Hawley JA (2007). The molecular bases of training adaptation. Sports Med.

[17] Snowling NJ, Hopkins WG (2006). Effects of different modes of exercise training on glucose control and risk factors for complications in type 2 diabetic patients: a meta-analysis. Diabetes Care.

[18] Krawetz SA, Kruger A, Lalancette C, Tagett R, Anton E, Draghici S (2011). A survey of small RNAs in human sperm. Hum Reprod.

[19] Castella S, Bernard R, Corno M, Fradin A, Larcher JC (2015). Ilf3 and NF90 functions in RNA biology. Wiley Interdiscip Rev RNA.

[20] Iliopoulos D, Hirsch HA, Struhl K (2009). An epigenetic switch involving NF-kappaB, Lin28, Let-7 MicroRNA, and IL6 links inflammation to cell transformation. Cell.

[21] Jiang LQ, Franck N, Egan B, Sjogren RJ, Katayama M, Duque-Guimaraes D (2013). Autocrine role of interleukin-13 on skeletal muscle glucose metabolism in type 2 diabetic patients involves microRNA let-7. Am J Physiol Endocrinol Metab.

[22] Wang X, Cao L, Wang Y, Wang X, Liu N, You Y (2012). Regulation of let-7 and its target oncogenes (review). Oncol Lett.

[23] Zhu H, Shyh-Chang N, Segre AV, Shinoda G, Shah SP, Einhorn WS (2011). The Lin28/let-7 axis regulates glucose metabolism. Cell.

[24] Kurotaki N, Imaizumi K, Harada N, Masuno M, Kondoh T, Nagai T (2002). Haploinsufficiency of NSD1 causes Sotos syndrome. Nat Genet.

[25] Lane C, Milne E, Freeth M (2017). Characteristics of autism Spectrum disorder in Sotos syndrome. J Autism Dev Disord.

[26] Falaleeva M, Welden JR, Duncan MJ, Stamm S. C/D-box snoRNAs form methylating and non-methylating ribonucleoprotein complexes: Old dogs show new tricks. Bioessays. 2017;39(6). 10.1002/bies.201600264 PMC5586538 28505386

[27] Ono M, Scott MS, Yamada K, Avolio F, Barton GJ, Lamond AI (2011). Identification of human miRNA precursors that resemble box C/D snoRNAs. Nucleic Acids Res.

[28] Cassidy SB, Schwartz S (1998). Prader-Willi and Angelman syndromes. Disorders of genomic imprinting. Medicine (Baltimore).

[29] Reimand J, Arak T, Adler P, Kolberg L, Reisberg S, Peterson H (2016). G:profiler-a web server for functional interpretation of gene lists (2016 update). Nucleic Acids Res.

[30] Denham J, O'Brien BJ, Harvey JT, Charchar FJ. Genome-wide sperm DNA methylation changes after 3 months of exercise training in humans. Epigenomics. 2015;8(2):307-8. 10.2217/epi.15.29 25864559

[31] Tang WW, Dietmann S, Irie N, Leitch HG, Floros VI, Bradshaw CR (2015). A unique gene regulatory network resets the human germline epigenome for development. Cell.

[32] Feinberg JI, Bakulski KM, Jaffe AE, Tryggvadottir R, Brown SC, Goldman LR (2015). Paternal sperm DNA methylation associated with early signs of autism risk in an autism-enriched cohort. Int J Epidemiol.

[33] Bailey TL, Boden M, Buske FA, Frith M, Grant CE, Clementi L (2009). MEME SUITE: tools for motif discovery and searching. Nucleic Acids Res.

[34] Pattamaprapanont P, Garde C, Fabre O, Barres R (2016). Muscle contraction induces acute hydroxymethylation of the exercise-responsive gene Nr4a3. Front Endocrinol (Lausanne).

[35] Liao Y, Smyth GK, Shi W (2013). The subread aligner: fast, accurate and scalable read mapping by seed-and-vote. Nucleic Acids Res.

[36] Liao Y, Smyth GK, Shi W (2014). Feature Counts: an efficient general purpose program for assigning sequence reads to genomic features. Bioinformatics.

[37] Kozomara A, Griffiths-Jones S (2014). miRBase: annotating high confidence microRNAs using deep sequencing data. Nucleic Acids Res.

[38] Zhang P, Si X, Skogerbo G, Wang J, Cui D, Li Y (2014). piRBase: a web resource assisting piRNA functional study. Database (Oxford).

[39] Karolchik D, Hinrichs AS, Furey TS, Roskin KM, Sugnet CW, Haussler D (2004). The UCSC table browser data retrieval tool. Nucleic Acids Res.

[40] Sarkar A, Maji RK, Saha S, Ghosh Z (2014). piRNAQuest: searching the piRNAome for silencers. BMC Genomics.

[41] Krueger F, Andrews SR (2011). Bismark: a flexible aligner and methylation caller for Bisulfite-Seq applications. Bioinformatics.

[42] Hebestreit K, Dugas M, Klein HU (2013). Detection of significantly differentially methylated regions in targeted bisulfite sequencing data. Bioinformatics.

[43] Machanick P, Bailey TL (2011). MEME-ChIP: motif analysis of large DNA datasets. Bioinformatics.

