# Supplementary figures and images for: Endurance training remodels sperm-borne small RNA expression and methylation at neurological gene hotspots
Source: Clin Epigenetics. 2018 Jan 25;10:12. doi: 10.1186/s13148-018-0446-7 (PMC5785820; doi:10.1186/s13148-018-0446-7)

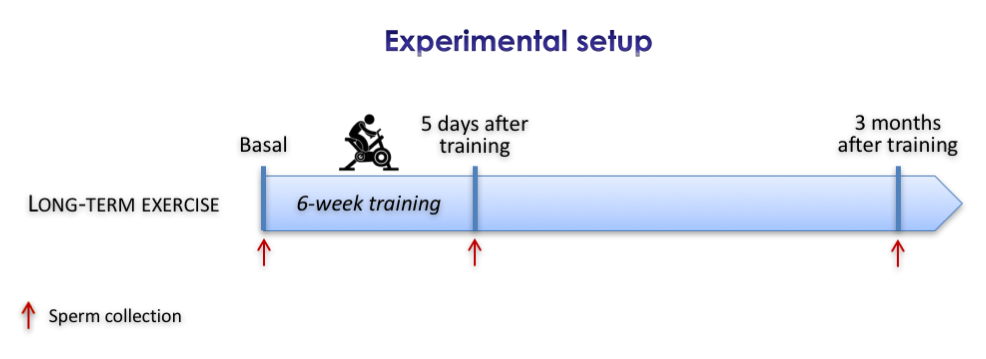

Supplement: Supplementary file 1 — Overview of the experimental setup. (TIFF 1386 kb) [file 13148_2018_446_MOESM1_ESM.tiff]

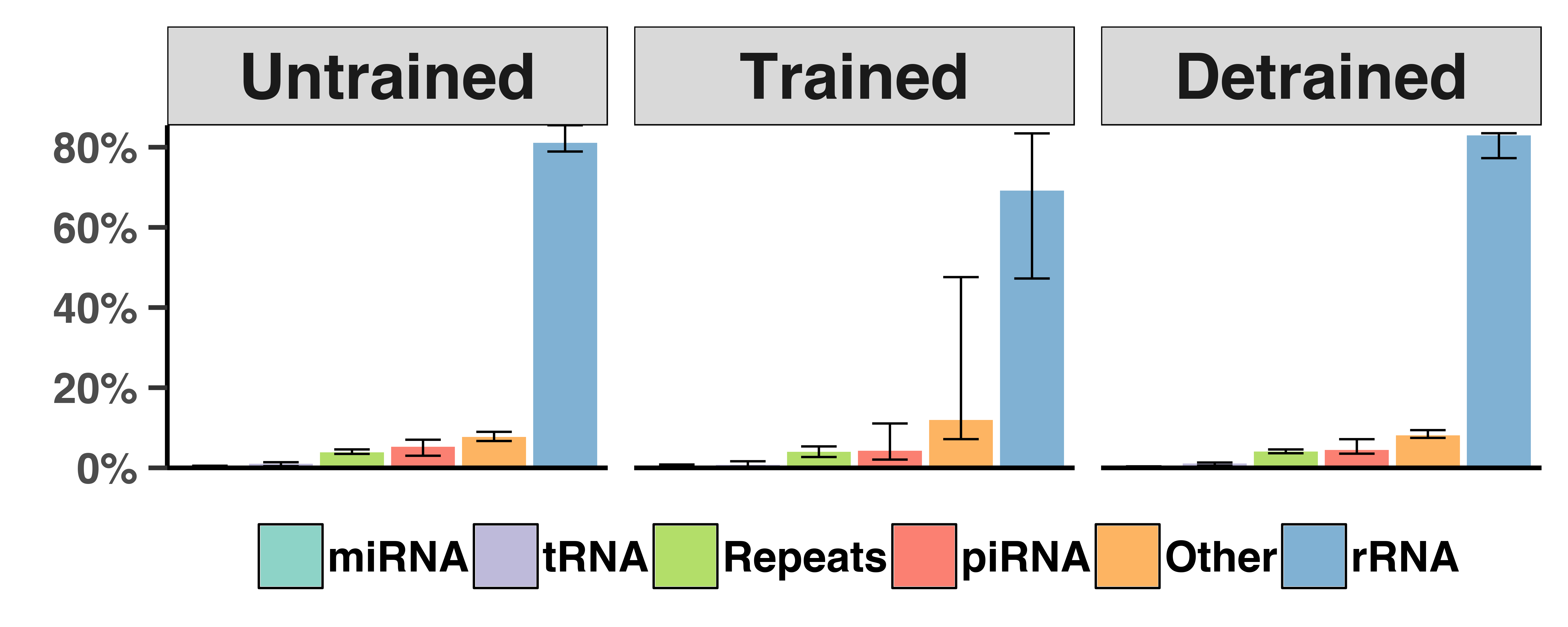

Supplement: Supplementary file 2 — Median abundance of all sRNA subtypes at the three different time points, height is median error bars are from lowest to highest observation. (TIFF 285 kb) [file 13148_2018_446_MOESM2_ESM.tiff]

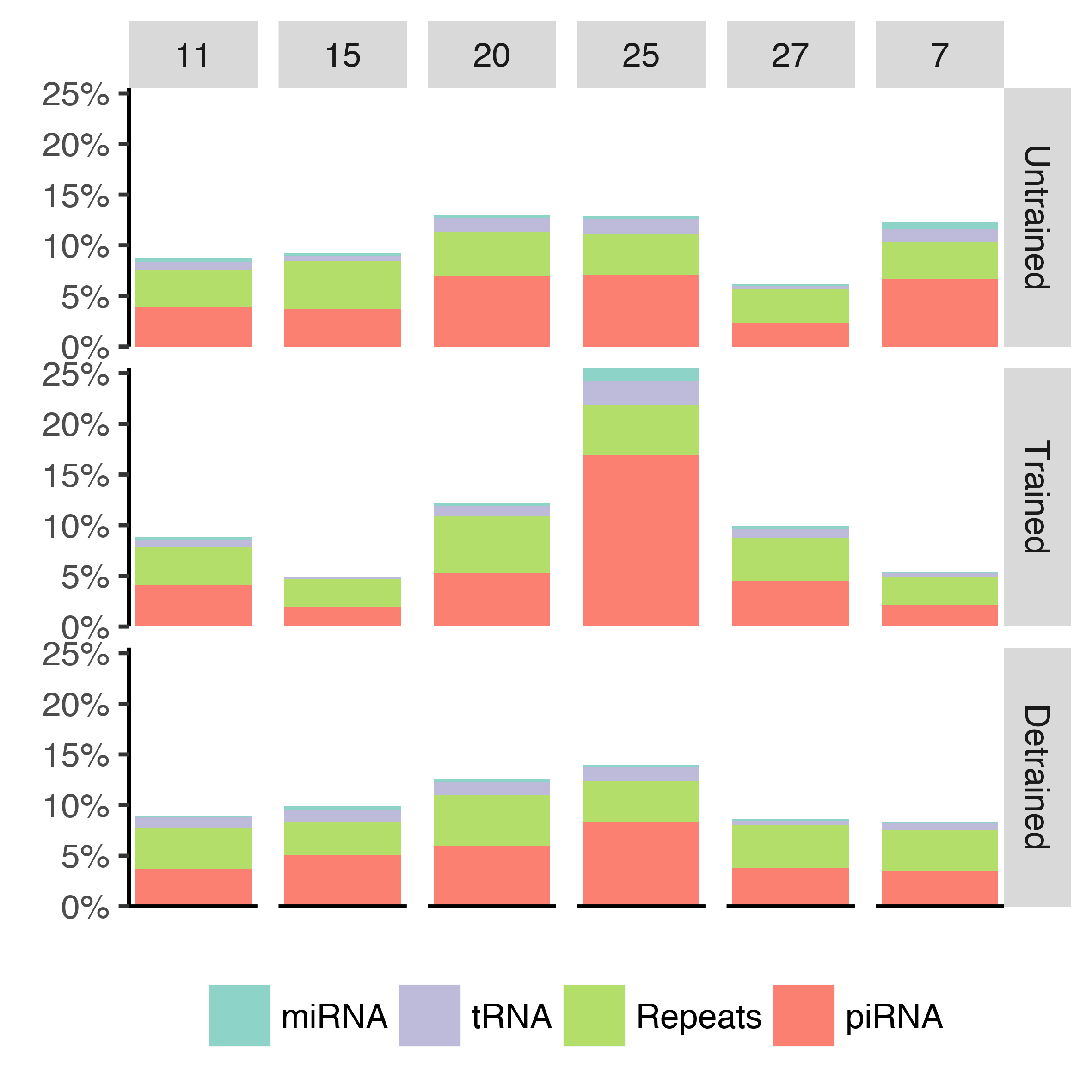

Supplement: Supplementary file 3 — Observed abundance of selected sRNA subtypes at the three different time points, columns represent different participants. (TIFF 189 kb) [file 13148_2018_446_MOESM3_ESM.tiff]

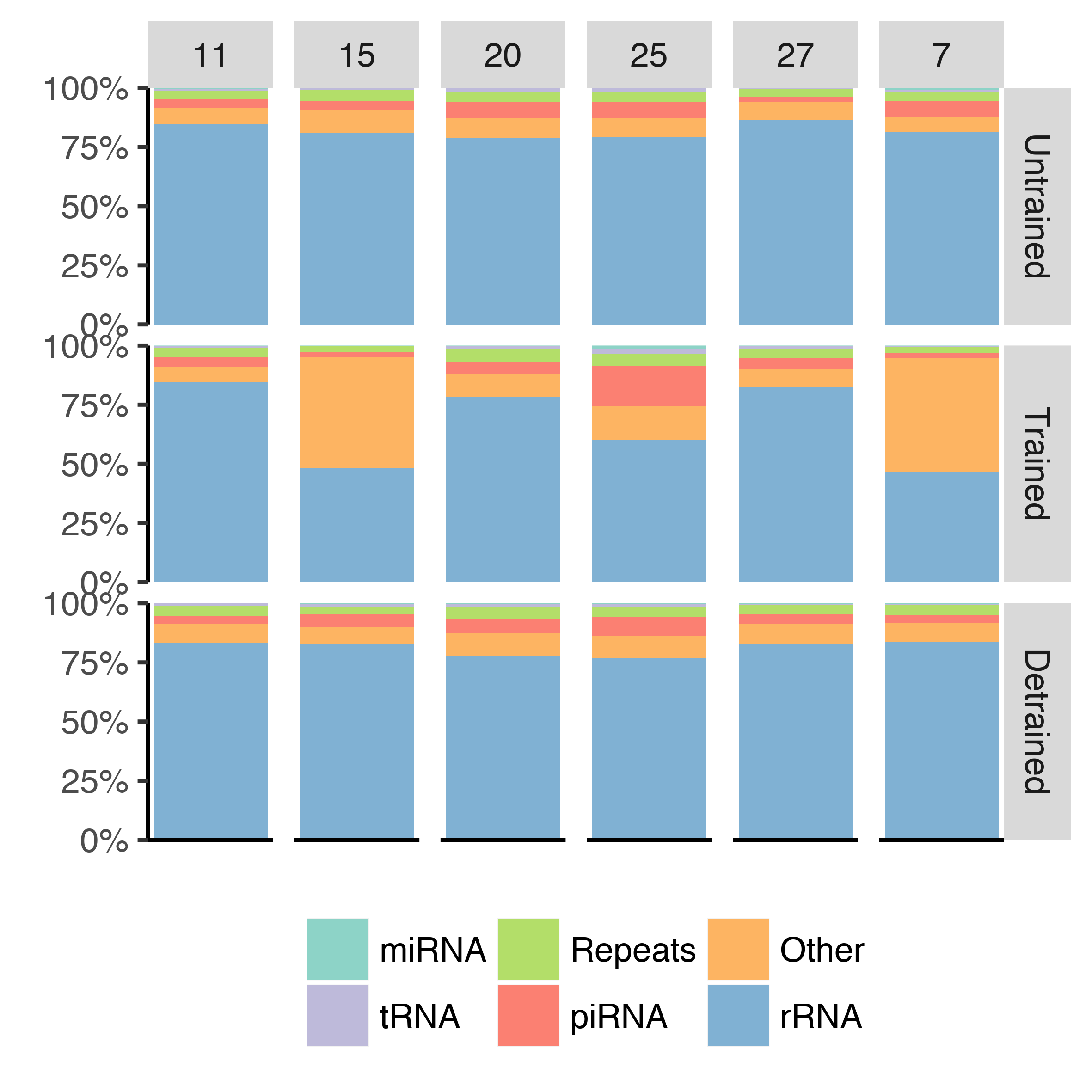

Supplement: Supplementary file 4 — Observed abundance of all sRNA subtypes at the three different time points, columns represent different participants. (TIFF 218 kb) [file 13148_2018_446_MOESM4_ESM.tiff]

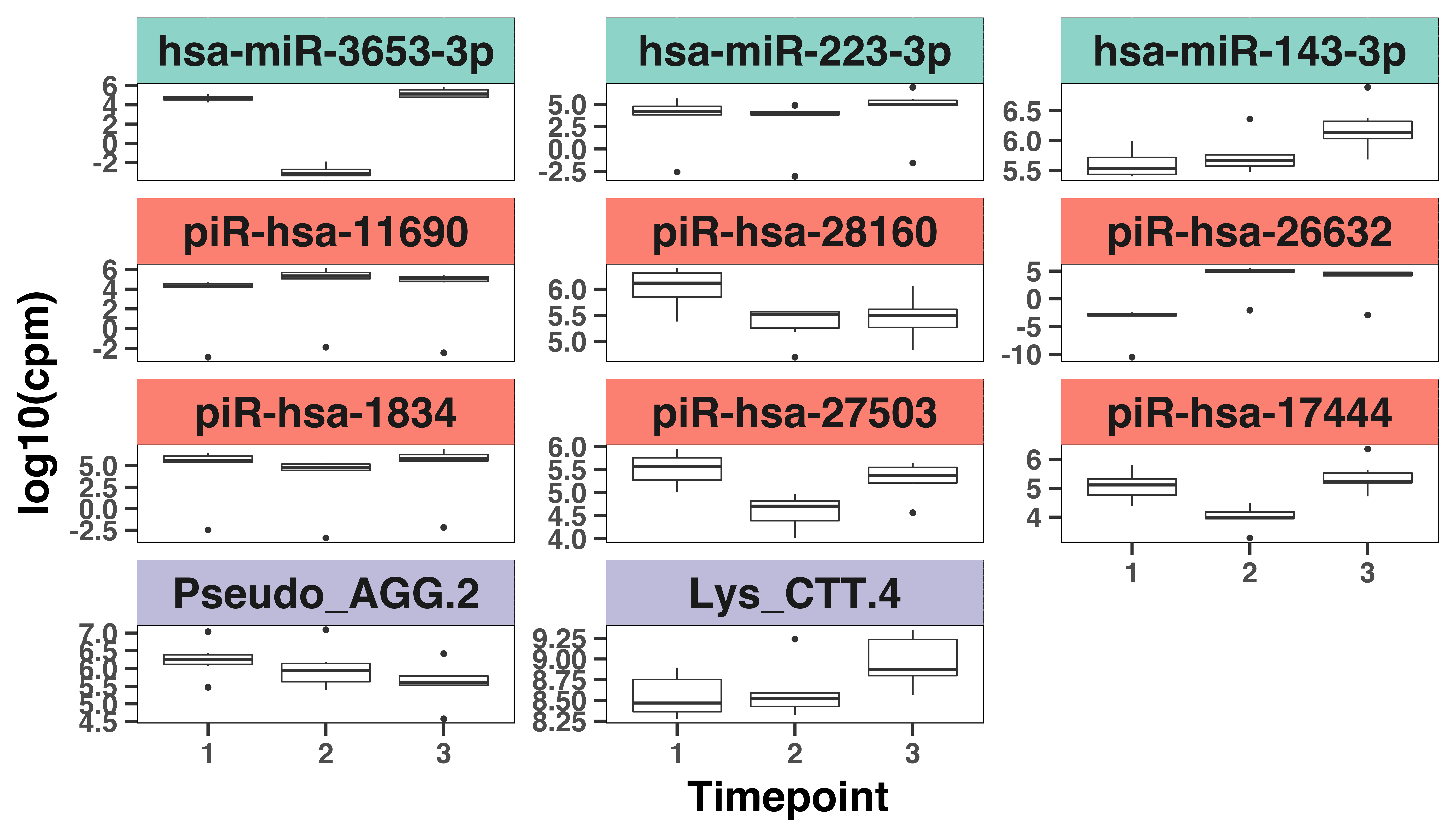

Supplement: Supplementary file 5 — Boxplot of the expression levels of selected subsets of sRNA (miRNA, green; tRNA, red; piRNA, purple) are presented at the three different time points for each individual. Data are presented as log-transformed sequence reads per million (1 = Untrained, 2 = Trained, 3 = Detrained). (TIFF 973 kb) [file 13148_2018_446_MOESM5_ESM.tiff]

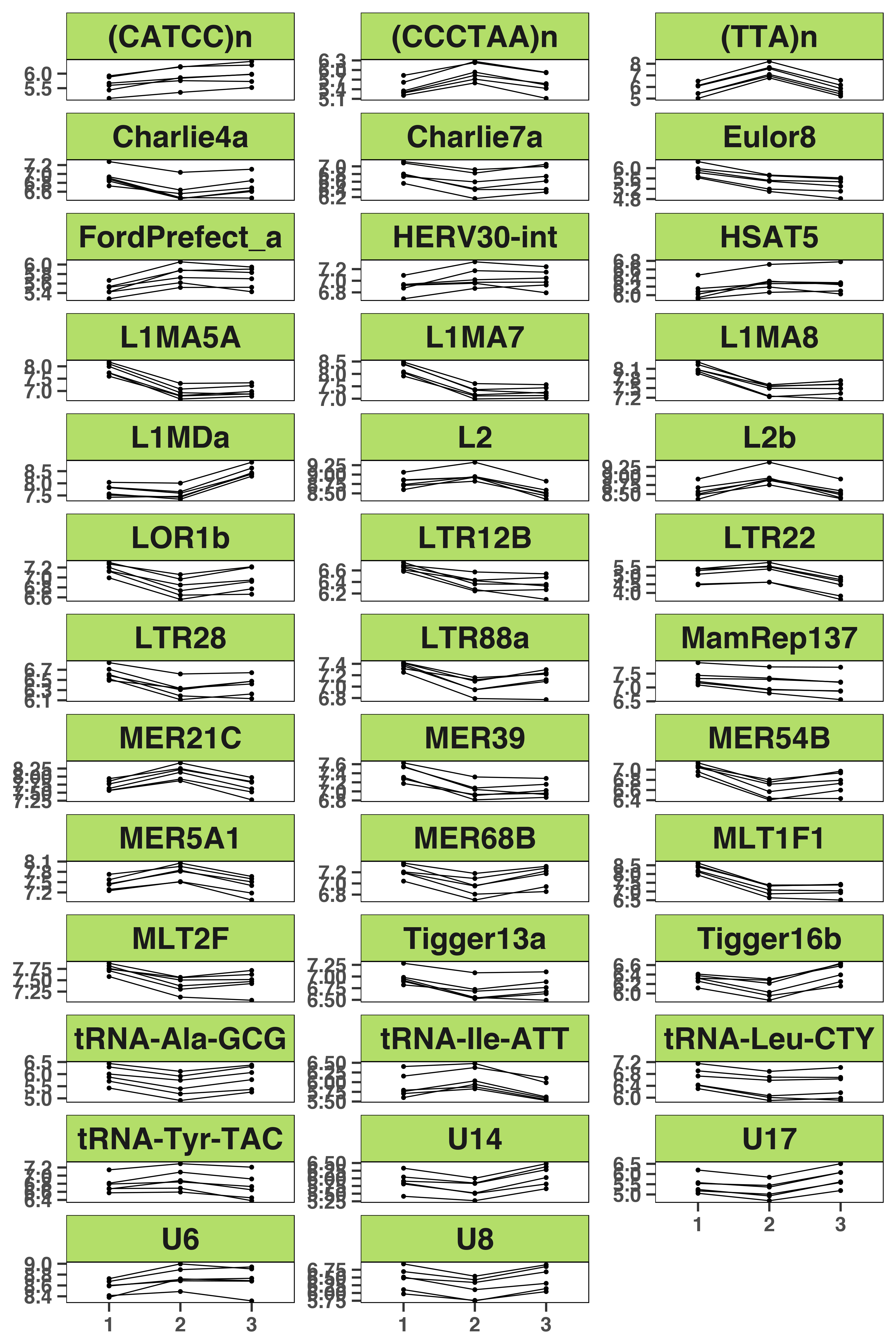

Supplement: Supplementary file 8 — The expression levels of selected subsets of Repetitive Elements are presented at the three different time points for each individual. Data are presented as log-transformed sequence reads per million (1 = Untrained, 2 = Trained, 3 = Detrained). (TIFF 1371 kb) [file 13148_2018_446_MOESM8_ESM.tiff]

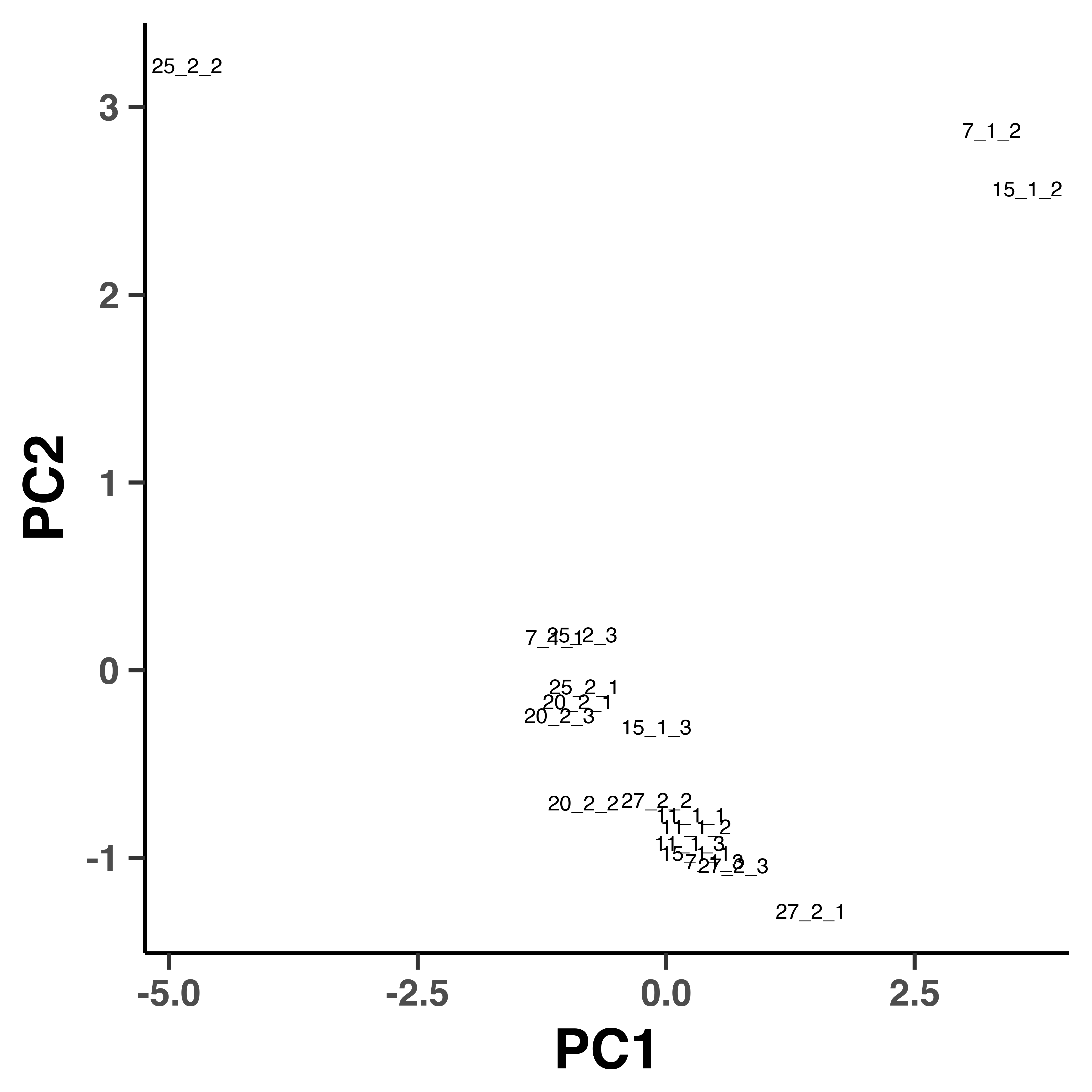

Supplement: Supplementary file 10 — PCA plot of the samples based on sncRNA distribution. The three samples 25_2_2, 7_1_2 and 15_1_2 were investigated as possible outliers, but no reason to exclude them could be found. (TIFF 220 kb) [file 13148_2018_446_MOESM10_ESM.tiff]

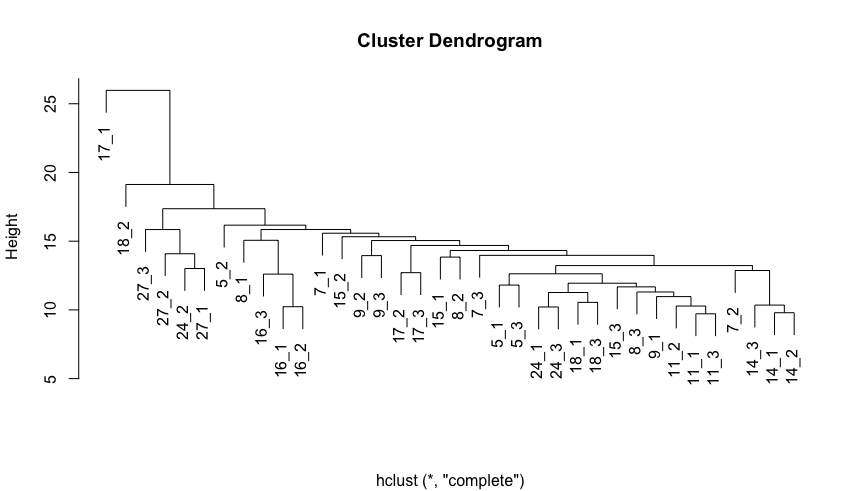

Supplement: Supplementary file 13 — Hierarchical clustering of samples based on estimated methylation across all covered CpGs. Sample 17_1 was sequenced less deeply and did not appear to be an outlier. (TIFF 1655 kb) [file 13148_2018_446_MOESM13_ESM.tiff]
